# Do LGTB people desire to have children? A Brazilian cross-sectional survey

**DOI:** 10.5935/1518-0557.20240094

**Published:** 2025

**Authors:** Rejane Casare, Kadija Chrisostomo, Najila Sandrin, Henrique Chrisostomo, Renato Nisihara

**Affiliations:** 1 Universidade Federal do Paraná, Curitiba, Brazil

**Keywords:** LGBT persons, children, assisted reproductive technique, prejudice

## Abstract

**Objective:**

To identify whether the LGBT community desires to start a family and the major challenges they experience, especially in regards to assisted reproductive technology (ART) procedures in Brazil.

**Methods:**

This cross-sectional study invited individuals Brazilian, identified themselves as cisgender, and homosexual (male and female); aged 18-60, the study used an online questionnaire distributed through various platforms.

**Results:**

Of the 698 respondents, mostly educated and Caucasian, 86.7% were female. While 69% expressed same-sex orientation, many were in long-term relationships. Family acceptance varied, with financial barriers (69.3%) and societal prejudices (73.1%) being significant concerns. Despite a desire for children (92% were childless), only 10% consulted infertility specialists, and 66.5% were unaware of ART legislation. Emotionally, 51.1% felt unprepared for parenthood. While societal acceptance of non-heteronormative families is perceived to be increasing (53%), unequal healthcare treatment (73.1%) and societal pressures (20% felt pressured towards adoption) remain prevalent.

**Conclusions:**

Over 90% of LGBT individuals contemplate having biological or adoptive children. There’s notable ignorance about legislation and ART. Financial hurdles in ART are significant but manageable with proper planning. Prejudice also strongly influences their lives.

## INTRODUCTION

According to Brazilian Institute of Geography and Statistics in 2019, 1.2% of Brazil’s adult population (approximately 1.8 million people) identified themselves as homosexual and 0.7% (1.1 million people) identified themselves as bisexual (IBGE, 2022a). Since 2013, same-sex marriage has been legalized in Brazil. (STF, 2011) Between 2013 and 2020, 59,620 same-sex marriages were registered (IBGE, 2022a). In 2013 these individuals gained access to assisted reproductive technology (ART) (CFM, 2013) and two years later (2015), the possibility to undergo the ROPA technique (Reception of Oocytes from Partner) (CFM, 2015). In 2022, the Federal Council of Medicine allowed the donation of gametes among relatives up to the fourth degree of kinship (parents, siblings, cousins and uncles) as long as consanguinity does not occur (CFM, 2022). This made it possible to conceive a child with two families’ genetics.

The LGBT community represents a large and increasing (Cross & Llewellyn, 2020), but understudied part of the ART population due to the predominant focus of ART and related research on the fertility of heterosexual couples (Defreyne *et al*., 2020). Therefore, existing research indicate that children´s development, adjustment, and psychological health are not affected by the gender identity or sexual orientation of parents (Raja *et al*., 2022). Despite having higher than average education levels, a significant number of same-sex couples are unaware of ART treatments that may provide biological children.

Although the LGBT community has obtained education, employment, financial resources and rights, they are still perceived as invisible by the majority, including the medical and scientific communities (Defreyne *et al*., 2020). The objective of this study is to identify whether the LGBT community desires to start a family and the major challenges they experience, especially in regards to ART procedures in Brazil.

## MATERIAL AND METHODS

### Ethical approval

This is a cross-sectional study approved by the local Research Ethics Committee under number 116476. An anonymous and voluntary online survey was available during November and December 2021.

### Participants

The questionnaire was entirely designed by the authors and converted into an online version via the Google Forms platform. The recruitment was conducted via social media and the questionnaire was shared through social networks, such as the Researcher’s Instagram and Facebook, LGBT groups and personal “blogs” of digital influencers in Brazil. The questionnaire was also shared by email and WhatsApp groups. All responses were anonymous and no participants were identified during the study survey.

Inclusion criteria consisted of individuals who were Brazilian, identified themselves as cisgender, and homosexual (male and female); with ages ranging from 18 to 60 years. An introductory text informed the study objectives. The participants were informed that the survey would take 15 to 20 minutes to be completed and that intimate questions would be addressed, such as questions related to sexuality. An orientation to answer the survey in a private space without interruptions was provided. After the participants read and agreed to participate, they signed the informed consent form online.

The following were exclusion criteria: incomplete questionnaires, duplicate responses and individuals who responded to the survey despite not meeting the inclusion criteria. We excluded individuals who identified themselves as transgender, gender variant, gender nonbinary, gender fluid, cross-dressers, etc.) to exclude bias in this study, since this is a diverse population with different needs for gender-affirming care, including the use of hormones or surgerywhich could affect their fertility (Defreyne *et al*., 2020).

### Questionnaire

A literature review did not yield studies with validated questions that addressed the desire of LGBT individuals to have children. Therefore, the authors elaborated a questionnaire in consultation with health professionals and LGBT individuals. The survey consisted of 63 questions divided into four sections.

**a. Section 1.** It included epidemiological questions such as age, education, ethnic background, income, number of children and health care access. Additionally, if the family is aware of sexual orientation and how they deal with it; who is aware of sexual orientation; if the individual is in a romantic relationship at the time of the study, for how long and what type of relationship (hetero-affective, homo-affective, single and fixed partner or open).

**b. Section 2.** Questions regarding children: if the individual has children, if the individual has a desire to have children and how the discovery of sexual orientation influenced this desire. Also, family planning: if the individual had already spoken to a doctor about their desire to have biological children.

**c. Section 3.** Questions regarding limitations, such as their emotional readiness to have children, the main barriers they face in relation to biological children and the greatest challenge that a non-traditional family faces. We also inquired about the idea of costs and accessibility of ART treatments.

**d. Section 4.** Questions focused on family acceptance: how this population perceives the acceptance of non-heteronormative families and biological or adopted children, and whether they felt compelled by society to adopt instead of trying to have biological children.

At the end of the questions, an informative text was attached, explaining all the options offered by ART and authorized by current Brazilian legislation. The author, who is certified in the field of ART, also provided a direct contact email address where participants could send any questions regarding the subject.

### Statistical analysis

Statistical analyzes were performed using the SPSS program. 17.0. The Kolmogorov-Smirnov and Shapiro-Wilk tests were used to assess data normality. Continuous variables were expressed as median and interquartile range (IQR), and were compared using the non-parametric Mann-Whitney test. Categorical variables were expressed in percentages and compared using the Fisher’s exact test or Chi-squared, as appropriate. *P* values less than 0.05 were considered statistically significant.

## RESULTS

The survey was answered by 776 individuals from all regions of Brazil. Seventy-eight surveys were excluded because the age limit fell outside the pre-defined range (<18 or >60 years old) and five surveys were excluded because the respondents did not reside in Brazil. The final sample available for evaluation consisted of 696 respondents, with a median age of 25 years (IQR=21-30 years).

Table 1 shows the main characteristics of the studied sample. In general, the sample had individuals with a higher level of education and Caucasian ethnic background. The majority of respondents were female (604/696; 86.7%). Among the participants, 68.9% identified their sexual orientation as same-sex. Of these, 73.9% were in a relationship with a single partner. The majority of the relationships were long-lasting, with 60.8% lasting between one and five years and 23.6% lasting over five years. Regarding the acceptance of sexual orientation by their family members, 37.4% responded that they had complete acceptance, while 39.1% responded that their families had reservations. Approximately 15% did not receive any family support and 6.4% hid their sexual orientation from their family. Regarding social interaction, 68.6% informed that they only disclosed their sexual orientation to their friends and 1.1% did not disclose it to anybody. It is noteworthy that 55.3% declare that they were not affiliated with any religion.

**Table 1:** Main characteristics of the studied sample (n=698).

Main characteristic	N	%
**Ethnicity/race** Afrodescendant Asian Caucasian Indigenous	106 36 520 36	15.1 5.2 74.5 5.2
Level of education Elementary High-School University Post-graduation	4 224 275 195	0.6 32.1 39.4 27.9
**Do you study or work in the health sciences field?** No Yes	464 234	66.5 33.5
**Personal income (minimal wage in Brazil = U$360.00)** Up to one minimal wage From 1 to 3 More than 3	193 266 225	28.2 38.9 32.9
**Religion** Catholic Spiritism Evangelical African matrix No religion	152 61 39 60 386	21.7 8.7 5.6 8.6 55.3
**Region of residence in Brazil** Central west Northeast North Southeast South	44 92 13 298 251	6.3 13.2 1.9 42.7 36.0
**Regarding sex at birth you have been designated as:** Man Woman Other	94 604 2	(13.5) (86.2) (0.3)
**Regarding your sexual orientation, you consider yourself:** Bisexual Homoaffective Pansexual	167 477 48	(24.2) (68.9) (6.9)
**How does your family deal with your sexual orientation?** Accepts and supports with reservations Accepts and supports without reservations It doesn’t accept my sexual orientation and doesn’t support my relationship Did not disclose the family Others	273 261 101 45 18	(39.1) (37.4) (14.5) (6.4) (2.6)
**Which people in your social life know about your sexual orientation?** Doctor/Physician Friends Family Co-workers I don’t tell anyone I don’t hide it from anyone	36 479 388 270 8 311	(5.2) (68.6) (55.6) (38.7) (1.1) (44.6)
**Are you currently in a relationship?** No Yes (open) Yes (Monogamous relationship)	154 28 516	(22.1) (4.0) (73.9)
**If yes. what is the length of the relationship:** Less than 1 year 1 - 5 years More than 5 years	85 332 129	(15.6) (60.8) (23.6)
**Your current relationship is:** Bisexual Homoaffective	15 523	(1.7) (98.3)

Table 2 provides data on the LGBT individuals’ desire to have children and whether the discovery of their sexual orientation changed the perspective on the subject. Approximately 92% do not have children, and only 6.3% stated that they did not wish to have children. For 48.1%, it makes no difference whether they have biological or adopted children, while 33.7% would like to have biological children.

**Table 2:** LGBT participants’ desire to have children and how the discovery of sexual orientation modified their opinion.

LGBT participants’ desire to have children and how the discovery of sexual orientation modified their opinion	N		%		
**Do you have children?** No Yes (adopted children) Yes (biological children)	642 7 49		92.0 1.0 7.0		
**Considering only your desire to have children, you would prefer:** Adoption I’m certain, I don’t intend to have (more) children Biological children Biological children and/or adoption. Is indifferent I don´t know	62 44 235 336 21		8.9 6.3 33.7 48.1 3.0		
**How did the discovery of your sexual orientation affect your desire to have biological children?** I wish I had biological children and would look for ART I wish I had biological children, but I would try natural insemination or ask friends/relatives for help I don’t know how I could have biological children I don’t know/ haven’t thought about it I’ve always thought about adopting	411 69 40 97 81		58.9 9.9 5.7 13.9 11.6		
**Have you ever talked to a doctor about your desire to have biological children?** No I’ve never talked to any doctor about it because I don’t feel comfortable. Yes, and he knew how to explain the possibilities Yes, but he did not know how to explain the possibilities	453 93 130 22		64.9 13.3 18.6 3.2		
**Did you know the Brazilian legislation about ART?** Yes No	234 464		32.5 66.5		

Among 92 men included, more than 73% of then (68/92) want to have children through adoption or biological, with 25/92 (7.1%) would like them to be biological. This value is significantly lower than observed in women (3.7%; *p*<0.0001).

Out of the total participants, 82 of 697 (11.7%) were over the age of 35. Among these older participants, 12 (14.6%, including 7 men) indicated that they did not plan to have children. In contrast, only 32 of the 615 participants younger than 35 (5.2%) expressed the same intention of not having biological or adopted children (*p*=0.0028). The discovery of sexual orientation did not change the desire to have children for 58.3% of participants. Only 10% consulted with a doctor specialized in infertility and 66.5% were unaware of the ART legislation.

Regarding difficulties and challenges reported by the studied sample, Table 3 shows that 51.1% of participants do not feel emotionally prepared to have children. The financial barrier was the most frequently reported (69.3%). For 83% of respondents, prejudice is the greatest challenge encountered by non-heteronormative families. There was no notable difference between men and women or in age in their responses on this issue. When asked about the value of ART, the majority of responders provided accurate answers. Despite the sample´s relatively low income, the majority (62.3%) reported that with financial planning, they could achieve their goal of having a child through ART. Only 13.8% of participants reported a lack of family support to have children. However, this factor had minimal impact on the desire to have children. Among the 100 participants who reported feeling a lack of family support, only three expressed that they did not want to have children.

**Table 3:** Difficulties and challenges reported by the studied sample (n=698).

Difficulties and challenges		N		%
**Do you feel emotionally prepared to have children?** No Yes		357 341		51.1 48.9
**What is your biggest barrier to having biological children?** Financial Lack of information Prejudice from others Lack of support from the partner Lack of family support Not being in a stable relationship I don’t see any barriers Age		484 108 104 29 96 106 95 34		69.3 15.5 14.9 4.2 13.8 15.2 13.6 4.9
**For you, what is the biggest challenge that a non-traditional family faces?** Lack of support from the partner Lack of family support I don’t see any challenges Others Prejudice		2 100 12 5 579		0.3 14.3 1.7 0.7 83.0
**Regarding the cost of treatment for ART, and according to your financial situation, do you believe they are:** Accessible Accessible with prior planning Inaccessible		15 416 267		2.1 59.6 38.3
**As for ART, how much do you believe the average cost of the procedure is:** Less than U$1,400 More than U$6,000 Between U$1,500 and U$6,000		13 79 666		1.9 11.3 86.8
**If ART would cost between U$1,500 and U$6,000, you would consider the procedure:** Accessible Accessible with prior planning Inaccessible		26 435 237		3.7 62.3 34.0

Table 4 shows data on the development of a non-heteronormative family. More than half (53%) of those interviewed believe that Brazilian society is more accepting of non-heteronormative families. However, if they require assistance to have children, 73.1% of those interviewed felt that they could be treated worse than heterosexual couples by the healthcare team. Only 15% believe that treatment is equal. In regards to non-heteronormative families, 63.6% responded that society does not accept non-heteronormative families, regardless of whether they have or do not have children, while 26.2% believe that society is more accepting of families without children. More than half of those interviewed (52.3%) believe that society is more accepting of same-sex couples who choose to adopt a child instead of trying to have biological children using ART. Approximately 20% reported feeling pressured (by family, friends or society) to try adoption instead of pursuing ART to have their biological children. Less than 4% of respondents believe that society accepts all non-heteronormative families, regardless of children.

**Table 4:** Formation of non-heteronormative families and acceptance by society.

	N	%
**Do you believe that Brazilian society is currently more accepting of non-heteronormative families non-heteronormative families?** No Yes	328 370	47.0 53.0
**Do you believe/feel that when homoaffective couples, seek help to try to have children, they are treated differently from heterosexuals?** It depends on the healthcare provider Different, need adequacy No (they are treated equally) I don’t know Yes, in a better way Yes, in a worse way	28 16 103 22 19 510	4.0 2.3 14.8 3.2 2.7 73.1
**Do you think society is more accepting of a homoaffective couple that:** Has children Has no children Accepts both options (with children and without children) Society does not accept either option	47 183 24 444	6.7 26.2 3.4 63.3
**Do you think society is more accepting of a homoaffective couple who chooses to adopt rather than try to have biological children with assisted reproduction?** No Yes	333 365	47.7 52.3
**Do you feel “pressured” (by family/friends/society) to adopt instead of trying for biological children?** No Yes	552 146	79.1 20.9

## DISCUSSION

Our study showed the challenges faced by the LGBT community in Brazil who desire to have children. Despite being increasing in the last five years (shown in primary care in England) (Cross & Llewellyn, 2020) and having higher educational status, they still have limited information about ART. Despite the majority of those studied being young (between 20 and 30 years old), more than 90% have the desire of having children, contrasting with Gato *et al*. (2020) findings, that LGBT people reported lower level of parenthood intentions when compared to their heterossexuals counterparts. In Brazil, 39.1% of women who become mothers between 30 and 39 years old in the state of São Paulo (Research by the State Data Analysis System Foundation - SEADE) (Boehm, 2020). It is important to note that older participants (over 35 years) expressed a lower desire to have children compared to younger participants. This trend is likely to be similar among heterosexual individuals.

In our study, the main barriers to having children were financial difficulties and societal prejudice, with no significant differences based on sex or age. Additionally, while family support is undoubtedly important, there was no significant difference in the desire to have children between those with and without such support.

In Brazil, it is legal to use gametes from oneself, one´s partner, an anonymous donor (fresh or frozen from a private bank) or a gamete from a relative up to the fourth degree through a formal, regulated healthcare provider. Therefore, the alternatives for a same-sex couple includes intrauterine insemination for female couples, or IVF from an anonymous or relative semen donor. In this case, they can choose: the regular IVF or ROPA. If none of them are able to become pregnant, they can choose a form of “surrogacy”. For male couples, IVF may be performed with an anonymous donor, donated gametes from a relative or a surrogate. It is worth noting that in Brazil surrogacy does not exist. So, for Brazilian masculine couple, is much more difficult - and expensive - having biological babies, since they will need a egg donor (relative or anonymous) and a - femalerelative until 4 degree) to generate the baby. Perhaps for these reasons, male participants in the study expressed a significantly lower desire to have children than women. The woman who will receive the embryo must be a relative up to the fourth degree and cannot be compensated (CFM, 2022).

This study sample was comprised of individuals with a higher level of education but lower income when compared with heterosexual people of the same age (IBGE, 2022a). This has been supported by other studies (Raja *et al*., 2022; Buchmueller & Carpenter, 2010). In this LGBT sample, approximately 3/4 are in a serious relationship with a single partner that has lasted a year or more. Despite their young age, more than 90% wish to have children, whether adopted or biologically using ART. In other studies, 60% of Italian LGBT people (Lasio *et al*., 2020) and 48% of North American lesbians reported wishing to have children compared to 51% of heterosexual individuals (Violette & Nguyen, 2023). In another study, van Houten *et al*. (2020) found that gay men and lesbian women expressed a similar strength of parenting intentions compared to their heterosexual peers.

Tate & Patterson, in the United States, found that lesbian and gay young adults with low parenthood aspirations were part of a general pattern of low expectations. Having more favorable parental relationships and more close friends were associated with greater likelihood of parenthood intentions (Tate & Patterson, 2019). In our sample, only 6.3% of individuals responded that they were certain they did not want to have children. Approximately 18% of US lesbians reported that they would be concerned if they were unable to have children while older Italian participants regretted not having them (Lasio *et al*., 2020).

The vast majority of individuals considered having children and could use ART to conceive; however, they have not consulted to a medical doctor about it. Providers should encourage disclosure by inquiring about sexual orientation since it’s been associated with regular health care use (Steele *et al*., 2006). Health professionals are less likely to ask lesbian patients about their desire regarding future pregnancy (Violette & Nguyen, 2023). In addition, many of them are unaware of the specific legislation and may be delayed in referral to reproductive specialists (Violette & Nguyen, 2023). This data shows the importance of greater dissemination of both the techniques for each group, as well as the Brazilian legislation on the subject.

The participants also responded that the main limitation in the use of ART is financial. This has also been reported in the USA (Violette & Nguyen, 2023). Other authors have reported that these barriers disproportionately affect the LGBT population (Ethics Committee of the American Society for Reproductive Medicine, 2021). In Brazil, few public services provide free ART, and they are primarily located in the Southeast and South regions, and with high wait times. Furthermore, ART is not covered by medical insurance and costs on average between US$ 1,400-6,000, making it exceedingly expensive for the majority of the population. However, it is noteworthy that 2/3 of participants responded that they could afford the costs provided they made financial planning. Over 30% of lesbians with medical insurance sought reproductive procedures compared to 10% of heterosexual individuals (Violette & Nguyen, 2023). Therefore, the LGBT population might be considered a “target” for reproductive clinics. A study published in 2017 reported that more than half of the fertility clinics that are members of the Society for Assisted Reproductive Technology in the US included LGBT-related content available on their websites (Wu *et al*., 2017). Predictive factors for having LGBT website content included location in northeastern and western regions and increasing the number of clinics (Jin & Dasgupta, 2016). According to online search results, only one of seven assisted reproduction clinics in Curitiba contains this information.

More than half of the participants (55.3%) stated they have no religion, a much higher percentage than the overall Brazilian population (14.3%) (IBGE, 2022b). A Portuguese study (Costa & Bidell, 2017) found that religiosity negatively predicted LGBT individuals’ intentions to raise children. This was not found in our study. In addition, a lack of family support was not frequently reported as a barrier to having children. This finding may be related to the fact that more than 70% of respondents stated that their families accept their sexual orientation and would be supportive if they had children.

For 83% of our participants, the greatest obstacle a non-traditional family faces is society’s prejudice. Although half of the sample perceived an improvement in the acceptance of non-heteronormative families by Brazilian society, they reported that a non-heteronormative couple intending to have a child will face worse treatment compared to a traditional family. A sensitive topic we asked about was if society was more accepting of the homoaffective couples who choose to adopt rather than try for biological children with ART. More than half answered “yes”. Adoption by same-sex couples is legally allowed in the United States, while certain states allow select agencies to refuse services to members of the LGBT community if the provision of these services conflicts with their religious beliefs. In 19 US states, there is no explicit protection against discrimination in adoption based on sexual orientation or gender identity (Movement Advancement Project, 2023). As Tate & Patterson (2019) noted: “LGBT and heterossexual young adults seem to be hoping for similar futures, but expecting different outcomes from one another”.

### Strengths and limitations

This study is limited by its cross-sectional design and low male participation. An online questionnaire was used in this study, and it had a nationwide reach, which would not have been feasible otherwise. The questionnaire was distributed through social media to people active in these media, stimulating interest and fostering discussions. It is possible that individuals who were already thinking about having children were more likely to complete the online questionnaire. On the contrary, because the questionnaire is anonymous and without any type of identification, respondents had the freedom to position themselves and be honest in their answers, avoiding embarrassment. The questionnaire was considered easy to understand, not requiring technical knowledge to complete and the terms were explained in each question.

The LGBT community still lacks significant attention from society regarding social demands. Non-heterosexual young Americans reported lower expectations than heterosexuals in areas such as: education, friendships, marriage, professional success, financial security and homeownership (Violette & Nguyen, 2023). There is no data on the Brazilian LGBT population, but it is likely that in a developing country, such numbers would be lower. Cultural and economic factors must significantly influence the results. Much of the existing research on this topic has focused on intentions, expectations, and desires regarding parenthood. Future studies could explore these aspects within our population.

## CONCLUSIONS

More than 90% of participants in the LGBT community consider the possibility having biological or adoptive children. However, such desire is significantly lower among men. A significant lack of knowledge regarding legislation and ART was observed. The financial barrier utilizing ART is substantial, but it may be overcome with planning. In addition, prejudice continues to have a significant impact on the lives of these individuals.

## References

[r1] Boehm C. (2020). Cresce índice de mulheres que se tornam mães dos 30 aos 39
anos.

[r2] Buchmueller T, Carpenter CS. (2010). Disparities in health insurance coverage, access, and outcomes
for individuals in same-sex versus different-sex relationships,
2000-2007. Am J Public Health.

[r3] CFM - Conselho Federal de Medicina (2013). Resolução Nº 2.013/13. Adota as normas éticas para
a utilização das técnicas de Reprodução
assistida.

[r4] CFM - Conselho Federal de Medicina (2015). Resolução Nº 2.121/2015. Adota as normas éticas
para a utilização das técnicas de
Reprodução assistida.

[r5] CFM - Conselho Federal de Medicina (2022). Resolução Nº 2.320/2022.

[r6] Costa PA, Bidell M. (2017). Modern Families: Parenting Desire, Intention, and Experience
Among Portuguese Lesbian, Gay, and Bisexual Individuals. J Fam Issues.

[r7] Cross H, Llewellyn CD. (2020). A decline in patient disclosure of heterosexuality in the English
General Practice Patient Survey: a longitudinal analysis of cross-sectional
data. Fam Pract.

[r8] Defreyne J, Van Schuylenbergh J, Motmans J, Tilleman KL, T’Sjoen GGR. (2020). Parental desire and fertility preservation in assigned female at
birth transgender people living in Belgium. Fertil Steril.

[r9] Ethics Committee of the American Society for Reproductive
Medicine (2021). Disparities in access to effective treatment for infertility in
the United States: an Ethics Committee opinion. Fertil Steril.

[r10] Gato J, Leal D, Coimbra S, Tasker F. (2020). Cross H, Llewellyn CD. A decline in patient disclosure of
heterosexuality in the English General Practice Patient Survey: a
longitudinal analysis of cross-sectional data. Fam Pract.

[r11] IBGE - Instituto Brasileiro de Geografia e
estatísticas (2022a). Em pesquisa inédita do IBGE, 2,9 milhões de adultos se
declararam homossexuais ou bissexuais em 2019.

[r12] IBGE - Instituto Brasileiro de Geografia e
estatísticas (2022b). Censo Demográfico. 2022.

[r13] Jin H, Dasgupta S. (2016). Disparities between online assisted reproduction patient
education for same-sex and heterosexual couples. Hum Reprod.

[r14] Lasio D, Lampis J, Spiga R, Serri F. (2020). Lesbian and Gay Individual Parenting Desires in Heteronormative
Contexts. Eur J Psychol.

[r15] Movement Advancement Project (2023). Equality Maps Foster Adopt. Laws.

[r16] Raja NS, Russell CB, Moravek MB. (2022). Assisted reproductive technology: considerations for the
nonheterosexual population and single parents. Fertil Steril.

[r17] Steele LS, Tinmouth JM, Lu A. (2006). Regular health care use by lesbians: a path analysis of
predictive factors. Fam Pract.

[r18] STF - Supremo Tribunal Federal (2011). Artigo Nº 1.723 do Código Civil. Reconhece união
estável homoafetiva.

[r19] Tate DP, Patterson CJ. (2019). Desire for Parenthood in Context of Other Life Aspirations Among
Lesbian, Gay, and Heterosexual Young Adults. Front Psychol.

[r20] Violette CJ, Nguyen BT. (2023). Expectations for family building, assisted reproduction, and
adoption among lesbians in the National Survey of Family Growth,
2017-2019. F S Rep.

[r21] van Houten JT, Tornello SL, Hoffenaar PJ, Bos HMW. (2020). Understanding Parenting Intentions Among Childfree Gay Men: A
Comparison With Lesbian Women and Heterosexual Men and Women. Front Psychol.

[r22] Wu HY, Yin O, Monseur B, Selter J, Collins LJ, Lau BD, Christianson MS. (2017). Lesbian, gay, bisexual, transgender content on reproductive
endocrinology and infertility clinic websites. Fertil Steril.

